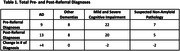# Implementation of Digital Cognitive Assessment for Early Detection of Cognitive Impairment: A Davos Alzheimer’s Collaborative Flagship Site

**DOI:** 10.1002/alz.087092

**Published:** 2025-01-09

**Authors:** Hisatomo Kowa, Kohei Morimoto

**Affiliations:** ^1^ Kobe University Graduate School of Health Sciences, Kobe Japan; ^2^ Kobe University Graduate School of Medicine, Kobe City Japan

## Abstract

**Background:**

In Japan, the number of patients with dementia is increasing and there is a shortage of human resources who can conduct detailed psychological examinations, and the development and implementation of new tools is essential for improving dementia care. Japan is one of seven sites across the globe focusing on healthcare system preparedness as part of the Davos Alzheimer’s Collaborative Early Detection flagship program. As part of this program, Japan implemented new tools including a digital cognitive assessment (DCA) and blood‐based biomarker (BBM) to increase rates of early detection of cognitive impairment.

**Methods:**

Patients 60+ years old were given a Mini‐Mental State Examination (MMSE), a common pen‐and‐paper test of cognitive function, and based on the score, primary care providers recorded a pre‐referral diagnosis. Patients who scored abnormal or borderline on the MMSE were then referred to a specialist center and completed a full work‐up including a clinical and psychological examination, a DCA (Cogstate Cognigram), blood test for reversible causes, imaging (i.e., MRI, perfusion SPECT), and a BBM (C_2_N PrecivityAD). Based on the results from the full clinical work‐up, the specialist provided a post‐referral diagnosis.

**Results:**

In the early detection program, the MMSE detected 46 total patients with cognitive impairment (MMSE scores ≥10 and <28), with 33 (72%) cognitively abnormal and 13 (28%) borderline scores. The number and type of pre‐referral diagnostic assessment made based on the MMSE scores and from the post‐referral diagnosis following the full clinical work‐up are provided in Table 1. There was good agreement between the initial and final diagnosis, with 8 total changes in diagnosis. The most notable changes came in the Alzheimer Disease diagnosis category where there was a net increase in 4 diagnoses of AD (2 were reversed and 6 new diagnoses of AD were added).

**Conclusion:**

Comprehensive assessments, including implementing new tools like a DCA and BBM, along with traditional clinical examination are necessary to make an accurate diagnosis. Initial findings from the Japan flagship site contribute to the global learnings to help speed and scale future innovations and developments in order to reach the right patients at the right time.